# Predictive value of the C-reactive protein-to-lymphocyte ratio for prognosis in heart failure patients with acute kidney injury

**DOI:** 10.3389/fphys.2026.1746567

**Published:** 2026-05-19

**Authors:** Supei Yin, Chengmei Shu, Dongli Huang, Zhihui Quan, Shuai Yang, Yin Wang

**Affiliations:** 1 Nephrology Department, Bishan Hospital of Chongqing, Bishan Hospital of Chongqing Medical University, Chongqing, China; 2 Department of Emergency Intensive Care Unit, Zhuhai People’s Hospital (The Affiliated Hospital of Beijing Institute of Technology, Zhuhai Clinical Medical College of Jinan University), Zhuhai, China; 3 Bishan Hospital of Chongqing, Bishan Hospital of Chongqing Medical University, Chongqing, China

**Keywords:** acute kidney injury, congestive heart failure, C-reactive protein, C-reactive protein to lymphocyte ratio, prognostic marker

## Abstract

**Background:**

The prognostic value of the C-reactive protein-to-lymphocyte ratio (CLR) in critical and inflammatory diseases is increasingly recognized. Nonetheless, its role in risk stratification for patients with congestive heart failure (CHF) who develop acute kidney injury (AKI) has not been thoroughly investigated. Research indicates that cardiorenal syndrome is mediated by a cytokine storm causing cardiac and renal injury, the extent of which can be quantified by the CLR and is significantly associated with adverse outcomes such as mortality and cardiovascular events. This study aims to elucidate the relationship between CLR and mortality in this patient population.

**Methods:**

Leveraging data from the MIMIC-IV database, we identified patients diagnosed with both CHF and AKI. We employed Cox models to gauge the link between CLR and near- and long-term (30-day/365-day) all-cause mortality, supplemented by Kaplan-Meier analysis across CLR quartiles. The non-linear dynamics of this relationship were mapped with restricted cubic splines (RCS). The model’s predictive performance was appraised using ROC curves, and potential effect modifiers were probed through comprehensive subgroup analyses to elucidate the interplay of key factors.

**Results:**

Among 1605 patients, higher CLR quartiles were associated with significantly worse survival by Kaplan–Meier analysis (log-rank P < 0.001). After full multivariable adjustment, the risk of 365-day mortality increased progressively across CLR quartiles (Q2: HR 1.96 [1.33–2.90]; Q3: 2.19 [1.49–3.21]; Q4: 3.78 [2.63–5.43]; P-trend < 0.001). Similar trends were observed for 30-day mortality (Q4: 4.32 [2.19–8.53]; P-trend < 0.001), although the HRs for Q2 and Q3 did not reach statistical significance. Fully adjusted RCS confirmed linear LnCLR–mortality associations for 30−day (P for nonlinearity = 0.352) and 365−day outcomes (P for nonlinearity = 0.197). ROC analysis demonstrated fair discriminative ability of CLR for 30-day (AUC: 0.713) and 365-day mortality (AUC: 0.675). Subgroup analyses confirmed consistent prognostic effects (all P for interaction > 0.05).

**Conclusion:**

CLR independently is associated with both short- and long-term mortality in CHF-AKI patients, supporting its clinical utility for risk stratification in this high-risk population.

## Introduction

The clinical trajectories of congestive heart failure (CHF) and acute kidney injury (AKI) represent critical junctures in cardiorenal axis decompensation, constituting major contributors to global healthcare challenges (Hoste et al., 2018; McDonagh et al., 2022; Peerapornratana et al., 2019). Contemporary epidemiology reveals CHF afflicts approximately 64 million individuals worldwide, with advanced-stage five-year mortality exceeding 50%, while 20-30% of hospitalized CHF patients develop AKI complications that tripled mortality risk compared to baseline (Hoste et al., 2018; McDonagh et al., 2022). This bidirectional organ crosstalk, pathologically manifested as cardiorenal syndrome (CRS), operates through three interlinked pathways: (i) hemodynamic instability provoking end-organ hypoperfusion, (ii) neuroendocrine hyperactivity disrupting fluid-electrolyte homeostasis, and (iii) cytokine storm-mediated processes perpetuating multiorgan injury cascades (Rangaswami et al., 2019). Notwithstanding paradigm-shifting therapeutic innovations, acute kidney injury complicating congestive heart failure (CHF-AKI) patients comorbidity continues to demonstrate dismal prognostic outcomes, highlighting an urgent imperative for discovering discriminatory biomarkers that enable pre-symptomatic risk stratification and precision-guided clinical decision-making.

The convergent pathomechanisms of CHF and AKI fundamentally involve dysregulated inflammatorycascades and immune homeostasis disruption. Heightened systemic levels of pleiotropic cytokines(e.g., IL-6, TNF-α) drive maladaptive myocardial fibrosis and nephron degeneration, whilechronic inflammation-associated lymphopenia reflects progressive erosion of adaptiveimmunocompetence, predisposing to opportunistic infections (Adamo et al., 2020; Vakhshoori et al., 2023). The C-reactive protein (CRP)/lymphocyte ratio (CLR) operationalizes this pathophysiological interplay, quantifying both systemic inflammation (CRP) and immunocompetence (lymphocytes). Emerging as a transdiagnostic prognosticator, CLR elevation shows dose-dependent mortality associations across sepsis, oncological, and atherosclerotic cohorts (Demirkol et al., 1992; Liu et al., 2020; De Clercq et al., 2023). Illustratively, septic patients with CLR>89.2 demonstrated superior discriminatory capacity for 28-day mortality (AUROC = 0.78) versus isolated CRP quantification (Kang et al., 2019). In acute coronary syndromes, CLR independently predicted composite cardiovascular endpoints during hospitalization, validating its pan-pathological utility (Kalyoncuoglu et al., 2020).

However, the prognostic value of CLR as a universal marker in critical and inflammatory illnesses remains to be elucidated in patients with CHF-AKI. Previous research on CHF biomarkers predominantly concentrated on natriuretic peptides (e.g., BNP, NT-proBNP) and renal indicators (e.g., creatinine, cystatin C), which, despite their value, do not specifically address the inflammatory-immune interaction (Brann et al., 2024; Nwanonenyi et al., 2024). Considering the combined impact of inflammation and immunosuppression in CHF-AKI patients, CLR could provide distinct prognostic insights. Initial evidence indicates that CLR is associated with negative outcomes in chronic kidney disease and post-cardiac surgery AKI, yet its predictive efficacy within the CHF-AKI patients has not been confirmed (Luo et al., 2021; Chen et al., 2022). This study seeks to fill this research void by utilizing data from the MIMIC-IV database to explore CLR’s correlation with 30-day and 365-day mortality among CHF-AKI patients. We hypothesize that elevated CLR levels signify a maladaptive inflammatory-immune response, contributing to adverse clinical outcomes.

## Materials and methods

### Data resource

Data for this study were sourced from the Medical Information Mart for Intensive Care IV (MIMIC-IV, version 3.0), a substantial, publicly accessible database comprising details on 76,943 ICU admissions. It includes extensive records on patient demographics, comorbidities, laboratory values, medications, and clinical outcomes. Access to the database was authorized for the authors (Shuai Yang) under Record ID 61695048, in adherence with ethical standards for data usage. Patient confidentiality was ensured by the de-identification of all personally identifiable information. The MIMIC-IV database received approval from the Massachusetts Institute of Technology and the Beth Israel Deaconess Medical Center. The data is publicly available (in the MIMIC-III database, Record ID: 52795482), therefore, the ethical approval statement and the requirement for informed consent were waived for this study.

### Study design and participants

This investigation employed a retrospective observational cohort study design to evaluate the prognostic significance of the CLR in mortality risk stratification among individuals diagnosed with concurrent CHF and AKI. The study population was derived from electronic health records using standardized diagnostic codes (ICD-9/ICD-10) for both CHF and AKI. Inclusion criteria required participants to be ≥18 years with documented CLR measurements [The C-reactive protein (CRP) to lymphocyte ratio (CLR) is derived from the formula: serum CRP (mg/dL) divided by the absolute lymphocyte count (×10³/µL). For instance, a CRP level of 10 mg/dL and a lymphocyte count of 2.0 ×10³/µL results in a CLR of 5] obtained within 24 hours of intensive care unit admission) and complete mortality follow-up data at 30-day and 1-year intervals. To ensure statistical independence of observations and a consistent baseline of new-onset critical illness, we restricted our analysis to each patient’s first recorded ICU admission during the hospital stay. Subsequent ICU readmissions for the same patient were excluded.

Exclusion parameters involved Patients were excluded based on the following criteria: (1) those who were not admitted to the ICU for the first time; (2) those lacking essential laboratory data for CLR calculation (CRP or lymphocyte count), creatinine, or other variables required for AKI diagnosis; (3) those who were discharged or died within 24 hours of ICU admission; (4) pregnant patients; and (5) those lost to follow-up or with missing prognostic information. Following screening, eligible participants were enrolled into the analysis cohort. Participants were categorized into quartile groups according to admission CLR values, with stratification boundaries determined by distribution patterns across the study population. Patients were stratified into quartiles based on their CLR levels, which were calculated as the ratio of CRP (mg/dL) to lymphocyte count (cells/μL), measured within the first 24 hours of ICU admission.

### Data collection

Patient characteristics such as age, sex, weight, and comorbid conditions—includinghypertension, myocardial infarction, peripheral vascular disease, cerebrovascular disease, chronic pulmonary disease, diabetes, renal disease, malignant cancer, and liver disease—were extracted from the MIMIC-IV database. Laboratory parameters collected included serum hemoglobin, creatinine, blood urea nitrogen, and C-reactive protein (CRP), among others. In addition, ICU severity scores, including Acute Physiology Score III (APS III), Simplified Acute Physiology Score II (SAPS II), and the Oxford Acute Severity of Illness Score (OASIS), were recorded to quantify baseline illness severity. Treatment-related variables—specifically, corticosteroid use, vasoactive agent administration, and continuous renal replacement therapy (CRRT)—were also extracted as key indicators of therapeutic intensity and organ support. The identification and classification of AKI adhered to the 2012 Kidney Disease: Improving Global Outcomes (KDIGO) guidelines. These guidelines define AKI based on several criteria: an increase in serum creatinine (sCr) of ≥0.3 mg/dL (26.5 μmol/L) within 48 hours; a rise in sCr to ≥1.5 times the baseline that is known or presumed to have occurred within the preceding 7 days; or urine output less than 0.5 mL/kg/hour for a duration of 6 hours. In cases where the baseline sCr was not documented prior to ICU admission, the first recorded sCr level post-admission was used as the baseline for comparison. Variables with more than 20% missing data were excluded from the analysis. For the remaining variables, missing values were imputed using a random forest approach (**Supplementary Figure 1** presents a visualization of the proportion of missing values for all study variables.). To assess the robustness of our findings to different missing data handling strategies, we performed a complete-case analysis (CCA) as a sensitivity analysis, excluding patients with any missing data in the covariates included in the primary multivariable Cox model.

### Statistical analysis

Statistical analyses were conducted using R software (version 4.4.1), with a two-sided P-value of less than 0.05 considered statistically significant. Continuous data with a skewed distribution were presented as median (M) and interquartile range (Q_1_, Q_3_). The Wilcoxon test was employed to compare differences between groups for such data. Categorical data were expressed as counts and percentages (n (%)) and analyzed using the chi-square test or Fisher’s exact test, as appropriate. Survival rates within different CLR-defined groups for both 30-day and 365-day mortality were assessed using Kaplan-Meier survival analysis, with differences between groups evaluated using the log-rank test. To investigate the relationship between CLR levels and all-cause mortality over these periods, univariate and multivariate Cox proportional hazards regression models were utilized. Model 1 included CLR index alone, while Model 2 adjusted for variables such as age, weight, gender, language, marital status, and race. Model 3 (fully adjusted) included additional adjustments for comorbid conditions including hypertension, myocardial infarction, peripheral vascular disease, cerebrovascular disease, chronic pulmonary disease, diabetes, renal disease, malignant cancer, and liver disease; ICU severity scores (APS III, SAPS II, OASIS); and treatment-related variables (corticosteroid use, vasoactive agents, and continuous renal replacement therapy [CRRT]). In these models, the lowest quartile of the CLR index served as the reference group. The trend across quartiles was tested by treating the median value of each quartile as a continuous variable, and the corresponding hazard ratio (HR for trend) and P for trend are reported. In addition, CLR was analyzed as a continuous variable after natural logarithm (ln) transformation in the Cox proportional hazards models to estimate the hazard ratio per unit increase in LnCLR. To identify an optimal prognostic threshold of CLR for clinical interpretability, we performed receiver operating characteristic (ROC) curve analysis for 30-day and 365-day mortality, respectively. The optimal cut-off value was determined using the Youden index (maximum of sensitivity + specificity – 1). Based on this approach, patients were dichotomized into low CLR and high CLR groups using cut-off values of 86.415 for 30-day mortality and 47.885 for 365-day mortality. Multivariable Cox proportional hazards regression models were then re-fitted using this binary CLR classification, with the same sequential adjustment strategy as in the primary analysis (Models 1–3). These analyses are presented as sensitivity analyses to assess the robustness of the CLR–mortality association under an alternative categorization scheme.

To examine the dose-response relationship between CLR and the risk of mortality at 30 and 365 days, CLR was natural logarithm (ln)-transformed and incorporated into restricted cubic spline (RCS) Cox regression models with four knots placed at the 5th, 35th, 65th, and 95th percentiles of the distribution. Both unadjusted (crude) and fully adjusted RCS models were constructed. The fully adjusted models included the same covariates as in Model 3 (demographics, comorbidities, ICU severity scores, and treatment-related variables). For each model, the P for overall association and P for nonlinearity were calculated to assess the significance of the association and departure from linearity, respectively. Subgroup analyses were conducted to identify potential effect modifiers, with stratification by gender (male, female), age (<60 years or ≥60 years), diabetes status, and history of hypertension.

## Result

### Baseline characteristics

**Figure 1:**
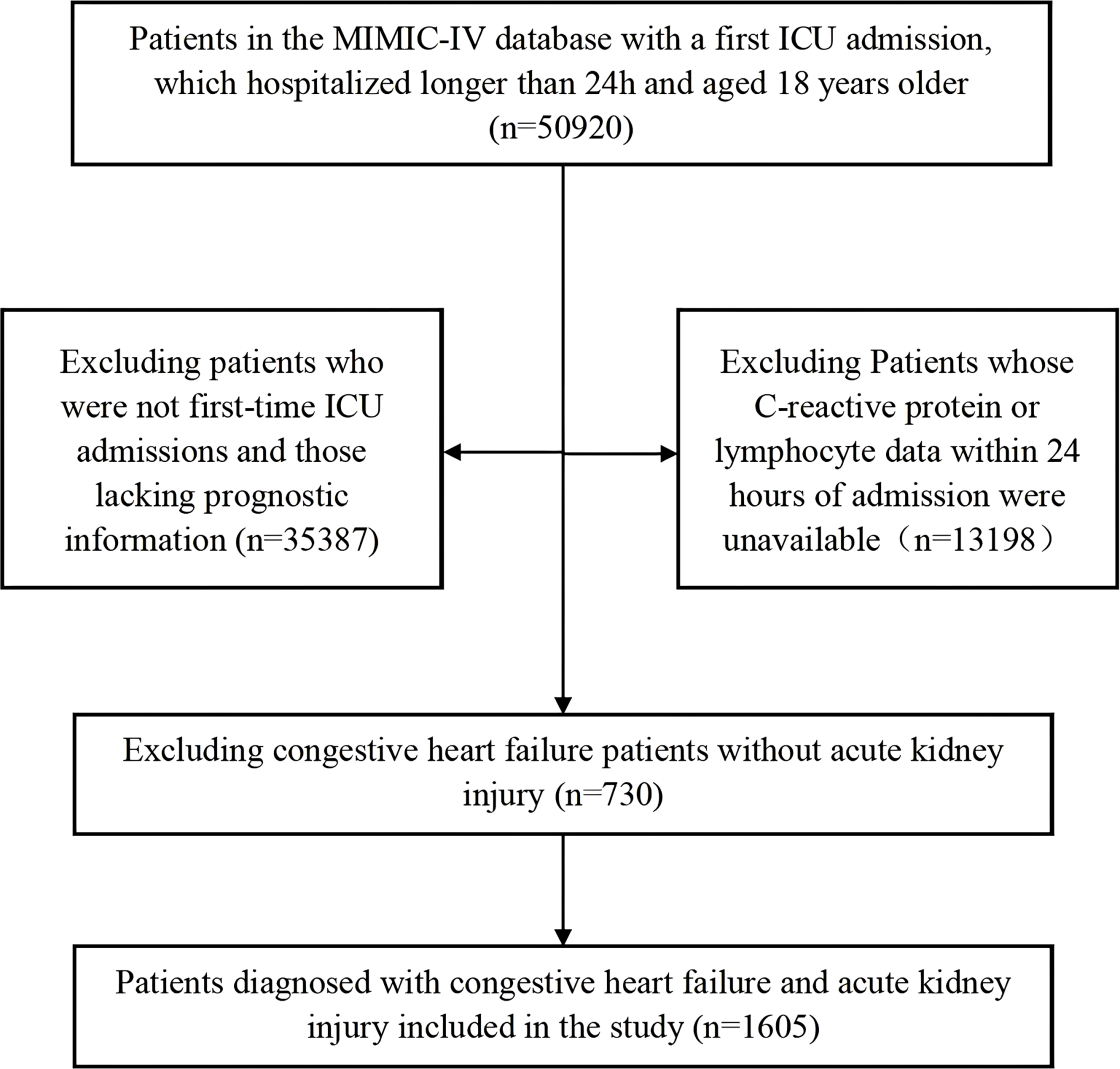
Flow of included patients through the trial.

This study included a total of 1605 participants, distributed into four CLR-based quartiles as depicted in **Figure 1**. Quartile 1 (Q1) included participants with CLR < 7.54, Quartile 2 (Q2) comprised those with 7.54 ≤ CLR < 37.12, Quartile 3 (Q3) included individuals with 37.12 ≤ CLR < 110.68, and Quartile 4 (Q4) consisted of participants with CLR ≥ 110.68. Statistically significant differences (P < 0.05) were observed among these groups concerning various clinical and laboratory parameters. These parameters included length of hospital stay, length of ICU stay, heart rate, systolic blood pressure (SBP), respiratory rate, temperature, SpO2, hematocrit, hemoglobin, anion gap, bicarbonate, blood urea nitrogen (BUN), calcium, chloride, creatinine, sodium, potassium, international normalized ratio (INR), prothrombin time (PT), partial thromboplastin time (PTT), Charlson Comorbidity Index, APS III score, SAPS II score, OASIS score, weight, CRP, lymphocyte count, CLR, and medical history of hypertension, dementia, renal disease, and sepsis, as detailed in **Table 1**.

**Table 1:** Baseline characteristics of four groups based on the quartiles of CLR index.

Variables	Total (n = 1605)	Q1 (n = 401)	Q2 (n = 401)	Q3 (n = 401)	Q4 (n = 402)	*P*
Los Hospital, M (Q_1_, Q_3_)	12.13 (7.23, 21.68)	9.79 (6.16,16.01)	11.95 (7.08,23.03)	12.79 (7.63,21.61)	15.36 (8.98,25.98)	**<.001**
Admission Age, M (Q_1_, Q_3_)	70.00 (61.00, 79.00)	70.00 (61.00,78.00)	70.00 (61.00,78.00)	70.00 (60.00,78.00)	71.00 (63.00,80.00)	0.094
Los Icu, M (Q_1_, Q_3_)	3.33 (1.85, 6.44)	2.83 (1.70,5.20)	3.07 (1.74,6.11)	3.35 (1.71,6.37)	4.17 (2.29,7.34)	**<.001**
Heart Rate Min, M (Q_1_, Q_3_)	70.00 (60.00, 81.00)	69.00 (59.00,79.00)	71.00 (62.00,81.00)	70.00 (60.00,81.00)	70.00 (61.00,81.00)	**0.049**
Heart Rate Max, M (Q_1_, Q_3_)	101.00 (88.00, 118.00)	96.00 (86.00,114.00)	101.00 (90.00,116.00)	100.00 (86.00,118.00)	103.00 (89.00,121.00)	**0.002**
Heart Rate Mean, M (Q_1_, Q_3_)	83.00 (74.00, 95.00)	82.00 (74.00,93.00)	84.00 (76.00,95.00)	83.00 (72.00,96.00)	83.50 (74.00,97.00)	0.097
Sbp Min, M (Q_1_, Q_3_)	88.00 (80.00, 97.00)	90.00 (82.00,99.00)	88.00 (81.00,97.00)	88.00 (80.00,98.00)	87.00 (79.00,95.75)	**<.001**
Sbp Max, M (Q_1_, Q_3_)	144.00 (130.00, 160.00)	144.00 (131.00,160.00)	145.00 (131.00,162.00)	143.00 (131.00,158.00)	143.00 (130.00,161.00)	0.602
Sbp Mean, M (Q_1_, Q_3_)	113.00 (105.00, 125.00)	116.00 (106.00,128.00)	114.00 (105.00,126.00)	112.00 (105.00,123.00)	112.00 (104.25,123.75)	**0.017**
Dbp Min, M (Q_1_, Q_3_)	45.00 (39.00, 52.00)	46.00 (39.00,52.00)	46.00 (41.00,53.00)	44.00 (38.00,52.00)	45.00 (39.00,52.00)	0.239
Dbp Max, M (Q_1_, Q_3_)	87.00 (74.00, 101.00)	87.00 (73.00,99.00)	87.00 (75.00,103.00)	86.00 (74.00,97.00)	88.00 (76.00,102.00)	0.101
Dbp Mean, M (Q_1_, Q_3_)	62.00 (55.00, 70.00)	62.00 (55.00,70.00)	62.00 (55.00,71.00)	62.00 (55.00,69.00)	62.00 (55.00,69.00)	0.687
Mbp Min, M (Q_1_, Q_3_)	59.00 (52.00, 65.00)	59.00 (54.00,66.00)	59.00 (54.00,65.00)	58.00 (52.00,65.00)	58.00 (50.00,65.00)	0.103
Mbp Max, M (Q_1_, Q_3_)	102.00 (91.00, 115.00)	102.00 (91.00,114.00)	102.00 (92.00,117.00)	100.00 (90.00,114.00)	102.00 (92.00,117.00)	0.164
Mbp Mean, M (Q_1_, Q_3_)	77.00 (71.00, 84.00)	77.00 (71.00,85.00)	77.00 (70.00,86.00)	76.00 (71.00,83.00)	77.00 (71.00,83.00)	0.457
Resp Rate Min, M (Q_1_, Q_3_)	13.00 (10.00, 15.00)	13.00 (10.00,15.00)	13.00 (10.00,15.00)	12.00 (10.00,15.00)	13.00 (11.00,15.00)	0.481
Resp Rate Max, M (Q_1_, Q_3_)	29.00 (25.00, 33.00)	28.00 (25.00,33.00)	28.00 (25.00,32.00)	28.00 (25.00,33.00)	30.00 (26.00,34.00)	**0.003**
Resp Rate Mean, M (Q_1_, Q_3_)	20.00 (17.00, 23.00)	20.00 (17.00,22.00)	20.00 (17.00,22.00)	19.00 (17.00,22.00)	20.00 (18.00,23.00)	**0.006**
Temperature Min, M (Q_1_, Q_3_)	36.44 (36.17, 36.67)	36.44 (36.13,36.61)	36.40 (36.17,36.66)	36.44 (36.11,36.61)	36.46 (36.28,36.67)	**0.023**
Temperature Max, M (Q_1_, Q_3_)	37.17 (36.94, 37.61)	37.17 (36.94,37.50)	37.17 (36.94,37.60)	37.20 (36.94,37.67)	37.22 (37.00,37.72)	0.175
Temperature Mean, M (Q_1_, Q_3_)	36.78 (36.60, 37.04)	36.77 (36.62,37.00)	36.76 (36.58,37.04)	36.78 (36.58,37.02)	36.83 (36.61,37.12)	0.067
Spo2 Min, M (Q_1_, Q_3_)	92.00 (89.00, 94.00)	92.00 (90.00,94.00)	92.00 (90.00,94.00)	92.00 (89.00,94.00)	91.00 (88.00,93.00)	**<.001**
Spo2 Max, M (Q_1_, Q_3_)	100.00 (99.00, 100.00)	100.00 (100.00,100.00)	100.00 (100.00,100.00)	100.00 (99.00,100.00)	100.00 (99.00,100.00)	0.311
Spo2 Mean, M (Q_1_, Q_3_)	97.00 (95.00, 98.00)	97.00 (96.00,98.00)	97.00 (95.00,98.00)	97.00 (95.00,98.00)	96.50 (95.00,98.00)	**0.005**
Hematocrit Min, M (Q_1_, Q_3_)	29.20 (25.00, 34.80)	30.10 (25.80,35.40)	29.50 (25.10,35.20)	28.40 (24.30,34.60)	29.00 (24.70,33.70)	**0.022**
Hematocrit Max, M (Q_1_, Q_3_)	33.90 (29.60, 38.60)	35.10 (30.70,39.60)	34.40 (30.10,38.80)	33.20 (28.70,37.90)	32.65 (28.52,37.80)	**<.001**
Hemoglobin Min, M (Q_1_, Q_3_)	9.40 (8.00, 11.20)	9.80 (8.30,11.50)	9.50 (8.10,11.50)	9.00 (7.80,11.00)	9.20 (7.80,10.80)	**<.001**
Hemoglobin Max, M (Q_1_, Q_3_)	10.90 (9.50, 12.40)	11.40 (9.90,12.90)	11.20 (9.60,12.60)	10.60 (9.10,12.20)	10.45 (9.20,12.00)	**<.001**
Platelets Min, M (Q_1_, Q_3_)	175.00 (123.00, 239.00)	172.00 (123.00,234.00)	176.00 (130.00,240.00)	180.00 (127.00,249.00)	170.50 (118.00,235.50)	0.173
Platelets Max, M (Q_1_, Q_3_)	213.00 (159.00, 281.00)	203.00 (156.00,277.00)	215.00 (166.00,286.00)	222.00 (163.00,292.00)	209.50 (146.00,270.75)	0.052
Wbc Min, M (Q_1_, Q_3_)	9.70 (7.10, 13.20)	9.50 (7.10,12.70)	9.90 (7.10,13.40)	9.70 (7.30,13.10)	9.60 (6.90,13.57)	0.582
Wbc Max, M (Q_1_, Q_3_)	13.20 (9.50, 18.80)	13.00 (9.70,19.30)	13.60 (9.40,18.50)	13.20 (9.80,18.70)	13.10 (9.22,18.48)	0.871
Aniongap Max, M (Q_1_, Q_3_)	16.00 (14.00, 19.00)	16.00 (13.00,19.00)	16.00 (14.00,19.00)	16.00 (14.00,19.00)	17.00 (14.00,20.00)	**0.018**
Aniongap Min, M (Q_1_, Q_3_)	13.00 (11.00, 16.00)	13.00 (11.00,15.00)	13.00 (11.00,16.00)	13.00 (11.00,16.00)	14.00 (11.00,16.00)	**0.029**
Bicarbonate Min, M (Q_1_, Q_3_)	21.00 (18.00, 24.00)	22.00 (19.00,24.00)	22.00 (19.00,24.00)	21.00 (18.00,24.00)	21.00 (17.00,24.00)	**0.002**
Bicarbonate Max, M (Q_1_, Q_3_)	24.00 (21.00, 27.00)	24.00 (22.00,27.00)	24.00 (22.00,27.00)	24.00 (21.00,27.00)	24.00 (20.00,26.75)	**0.002**
Bun Min, M (Q_1_, Q_3_)	25.00 (16.00, 41.00)	20.00 (14.00,31.00)	23.00 (15.00,36.00)	27.00 (16.00,45.00)	31.00 (18.25,50.00)	**<.001**
Bun Max, M (Q_1_, Q_3_)	30.00 (19.00, 49.00)	25.00 (17.00,37.00)	28.00 (18.00,45.00)	33.00 (20.00,53.00)	38.00 (24.00,59.00)	**<.001**
Calcium Min, M (Q_1_, Q_3_)	8.20 (7.80, 8.70)	8.30 (7.90,8.80)	8.30 (7.90,8.80)	8.20 (7.80,8.60)	8.20 (7.70,8.60)	**<.001**
Calcium Max, M (Q_1_, Q_3_)	8.60 (8.20, 9.10)	8.80 (8.30,9.20)	8.70 (8.30,9.10)	8.60 (8.10,9.10)	8.60 (8.20,9.00)	**<.001**
Chloride Min, M (Q_1_, Q_3_)	100.00 (96.00, 104.00)	101.00 (98.00,105.00)	100.00 (96.00,104.00)	100.00 (96.00,104.00)	99.00 (95.00,103.00)	**<.001**
Chloride Max, M (Q_1_, Q_3_)	104.00 (100.00, 108.00)	105.00 (101.00,109.00)	104.00 (99.00,108.00)	104.00 (100.00,108.00)	102.50 (98.00,107.00)	**<.001**
Creatinine Min, M (Q_1_, Q_3_)	1.30 (0.90, 2.10)	1.00 (0.80,1.60)	1.10 (0.90,1.90)	1.40 (0.90,2.50)	1.50 (1.00,2.80)	**<.001**
Creatinine Max, M (Q_1_, Q_3_)	1.50 (1.00, 2.60)	1.20 (0.90,2.00)	1.40 (1.00,2.30)	1.60 (1.10,3.00)	1.85 (1.20,3.60)	**<.001**
Glucose Min, M (Q_1_, Q_3_)	116.00 (98.00, 143.00)	113.00 (97.00,141.00)	116.00 (98.00,142.00)	119.00 (99.00,146.00)	118.00 (99.00,143.75)	0.381
Glucose Max, M (Q_1_, Q_3_)	154.00 (124.00, 211.00)	148.00 (119.00,216.00)	151.00 (125.00,207.00)	160.00 (128.00,215.00)	157.50 (129.00,209.00)	0.195
Sodium Min, M (Q_1_, Q_3_)	137.00 (134.00, 139.00)	137.00 (134.00,139.00)	137.00 (134.00,139.00)	137.00 (134.00,139.00)	136.00 (132.00,139.00)	**<.001**
Sodium Max, M (Q_1_, Q_3_)	139.00 (137.00, 142.00)	140.00 (137.00,142.00)	140.00 (137.00,142.00)	139.00 (137.00,142.00)	139.00 (136.00,142.00)	**0.009**
Potassium Min, M (Q_1_, Q_3_)	4.00 (3.60, 4.40)	4.00 (3.50,4.40)	4.00 (3.60,4.40)	4.00 (3.60,4.50)	4.00 (3.70,4.50)	**0.039**
Potassium Max, M (Q_1_, Q_3_)	4.60 (4.20, 5.20)	4.50 (4.10,5.00)	4.60 (4.20,5.10)	4.70 (4.30,5.30)	4.70 (4.20,5.30)	**0.009**
Inr Min, M (Q_1_, Q_3_)	1.30 (1.10, 1.50)	1.20 (1.10,1.40)	1.30 (1.10,1.40)	1.30 (1.10,1.50)	1.30 (1.20,1.60)	**<.001**
Inr Max, M (Q_1_, Q_3_)	1.40 (1.20, 1.70)	1.40 (1.20,1.60)	1.40 (1.20,1.80)	1.40 (1.20,1.80)	1.50 (1.30,1.90)	**<.001**
Pt Min, M (Q_1_, Q_3_)	13.80 (12.40, 16.20)	13.30 (12.10,15.00)	13.80 (12.40,15.90)	13.90 (12.40,16.40)	14.50 (12.80,17.10)	**<.001**
Pt Max, M (Q_1_, Q_3_)	15.40 (13.30, 19.00)	15.00 (13.10,17.80)	15.30 (13.10,19.10)	15.60 (13.30,19.30)	15.80 (13.70,21.28)	**0.003**
Ptt Min, M (Q_1_, Q_3_)	29.90 (26.80, 34.50)	29.50 (26.80,33.50)	29.50 (26.60,34.30)	29.90 (26.60,34.60)	30.80 (27.22,36.10)	**0.030**
Ptt Max, M (Q_1_, Q_3_)	35.60 (29.90, 55.20)	35.10 (30.30,53.00)	35.40 (29.10,53.00)	35.40 (30.20,54.00)	37.55 (30.22,59.45)	0.236
Charlson Comorbidity Index, M (Q_1_, Q_3_)	7.00 (6.00, 9.00)	7.00 (5.00,8.00)	7.00 (6.00,9.00)	8.00 (6.00,9.00)	8.00 (6.00,10.00)	**<.001**
Apsiii, M (Q_1_, Q_3_)	49.00 (39.00, 65.00)	44.00 (35.00,57.00)	47.00 (36.00,62.00)	51.00 (40.00,67.00)	55.00 (44.00,76.00)	**<.001**
Sapsii, M (Q_1_, Q_3_)	38.00 (31.00, 47.00)	36.00 (28.00,43.00)	37.00 (31.00,44.00)	38.00 (32.00,48.00)	42.50 (34.00,51.00)	**<.001**
Oasis, M (Q_1_, Q_3_)	33.00 (27.00, 39.00)	31.00 (25.00,37.00)	33.00 (27.00,39.00)	33.00 (27.00,41.00)	35.00 (29.00,42.00)	**<.001**
Weight Admit, M (Q_1_, Q_3_)	87.20 (72.20, 104.00)	84.80 (70.00,99.60)	88.90 (74.10,104.30)	90.00 (72.00,108.20)	86.70 (71.73,102.57)	**0.028**
Crp, M (Q_1_, Q_3_)	47.10 (10.20, 114.60)	3.90 (1.60,6.80)	25.30 (14.10,43.70)	80.20 (54.00,119.50)	164.75 (109.70,212.12)	**<.001**
Lymphocytes Abs, M (Q_1_, Q_3_)	1.19 (0.73, 1.75)	1.59 (1.10,2.18)	1.42 (0.87,2.09)	1.22 (0.81,1.72)	0.68 (0.41,1.02)	**<.001**
CLR, M (Q_1_, Q_3_)	37.12 (7.54, 110.68)	2.47 (1.10,4.79)	17.67 (12.20,27.60)	65.78 (50.57,86.37)	211.91 (149.72,351.10)	**<.001**
Hospital Expire, n (%)						**<.001**
No	1477 (92.02)	393 (98.00)	376 (93.77)	375 (93.52)	333 (82.84)	
Yes	128 (7.98)	8 (2.00)	25 (6.23)	26 (6.48)	69 (17.16)	
Gender, n (%)						**<.001**
Male	916 (57.07)	191 (47.63)	235 (58.60)	230 (57.36)	260 (64.68)	
Female	689 (42.93)	210 (52.37)	166 (41.40)	171 (42.64)	142 (35.32)	
Language, n (%)						**0.006**
English	1402 (87.35)	330 (82.29)	358 (89.28)	357 (89.03)	357 (88.81)	
Non-English	203 (12.65)	71 (17.71)	43 (10.72)	44 (10.97)	45 (11.19)	
Marital Status, n (%)						0.777
Married	800 (49.84)	196 (48.88)	195 (48.63)	205 (51.12)	204 (50.75)	
Divorced	143 (8.91)	40 (9.98)	36 (8.98)	36 (8.98)	31 (7.71)	
Single	440 (27.41)	109 (27.18)	110 (27.43)	116 (28.93)	105 (26.12)	
Widowed	222 (13.83)	56 (13.97)	60 (14.96)	44 (10.97)	62 (15.42)	
Race, n(%)						0.398
White	980 (61.06)	242 (60.35)	257 (64.09)	239 (59.60)	242 (60.20)	
Black	289 (18.01)	79 (19.70)	72 (17.96)	74 (18.45)	64 (15.92)	
Other	336 (20.93)	80 (19.95)	72 (17.96)	88 (21.95)	96 (23.88)	
Hypertension, n (%)						**0.021**
No	319 (19.88)	84 (20.95)	81 (20.20)	94 (23.44)	60 (14.93)	
Yes	1286 (80.12)	317 (79.05)	320 (79.80)	307 (76.56)	342 (85.07)	
Myocardial Infarct, n (%)						0.172
No	1113 (69.35)	294 (73.32)	275 (68.58)	278 (69.33)	266 (66.17)	
Yes	492 (30.65)	107 (26.68)	126 (31.42)	123 (30.67)	136 (33.83)	
Peripheral Vascular Disease, n (%)						0.061
No	1323 (82.43)	346 (86.28)	325 (81.05)	333 (83.04)	319 (79.35)	
Yes	282 (17.57)	55 (13.72)	76 (18.95)	68 (16.96)	83 (20.65)	
Cerebrovascular Disease, n (%)						0.178
No	1375 (85.67)	352 (87.78)	331 (82.54)	344 (85.79)	348 (86.57)	
Yes	230 (14.33)	49 (12.22)	70 (17.46)	57 (14.21)	54 (13.43)	
Chronic Pulmonary Disease, n (%)						0.146
No	1119 (69.72)	292 (72.82)	264 (65.84)	276 (68.83)	287 (71.39)	
Yes	486 (30.28)	109 (27.18)	137 (34.16)	125 (31.17)	115 (28.61)	
Diabetes, n (%)						0.075
No	834 (51.96)	224 (55.86)	218 (54.36)	200 (49.88)	192 (47.76)	
Yes	771 (48.04)	177 (44.14)	183 (45.64)	201 (50.12)	210 (52.24)	
Renal Disease, n (%)						**<.001**
No	879 (54.77)	266 (66.33)	231 (57.61)	199 (49.63)	183 (45.52)	
Yes	726 (45.23)	135 (33.67)	170 (42.39)	202 (50.37)	219 (54.48)	
Malignant Cancer, n (%)						0.314
No	1477 (92.02)	374 (93.27)	373 (93.02)	368 (91.77)	362 (90.05)	
Yes	128 (7.98)	27 (6.73)	28 (6.98)	33 (8.23)	40 (9.95)	
Liver Disease, n (%)						0.534
No	1441 (89.78)	364 (90.77)	364 (90.77)	359 (89.53)	354 (88.06)	
Yes	164 (10.22)	37 (9.23)	37 (9.23)	42 (10.47)	48 (11.94)	
Sepsis, n (%)						**<.001**
No	627 (39.07)	191 (47.63)	172 (42.89)	158 (39.40)	106 (26.37)	
Yes	978 (60.93)	210 (52.37)	229 (57.11)	243 (60.60)	296 (73.63)	
Status 30, n (%)						**<.001**
No	1473 (91.78)	391 (97.51)	378 (94.26)	372 (92.77)	332 (82.59)	
Yes	132 (8.22)	10 (2.49)	23 (5.74)	29 (7.23)	70 (17.41)	
Status 365, n (%)						**<.001**
No	1237 (77.07)	363 (90.52)	322 (80.30)	308 (76.81)	244 (60.70)	
Yes	368 (22.93)	38 (9.48)	79 (19.70)	93 (23.19)	158 (39.30)	

Q1, CLR<7.54; Q2, 7.54≤CLR<37.12; Q3, 37.12≤CLR<110.68; Q4, CLR≥110.68.

M, Median; Q_1_, 1st Quartile; Q_3_, 3st Quartile.

ICU, Intensive Care Unit; SBP, Systolic Blood Pressure; DBP, Diastolic Blood Pressure; MBP, Mean Blood Pressure; Resp Rate, Respiration Rate; WBC, White blood cell; Bun, Blood Urea Nitrogen; INR, International Normalized Ratio; PT, Prothrombin Time; PTT, Partial Thromboplastin Time; CRP, C-reactive protein; CLR, C-reactive protein/lymphocyte ratio.

Bold values P < .005 indicates statistical significance.

### Study outcomes

**Figure 2:**
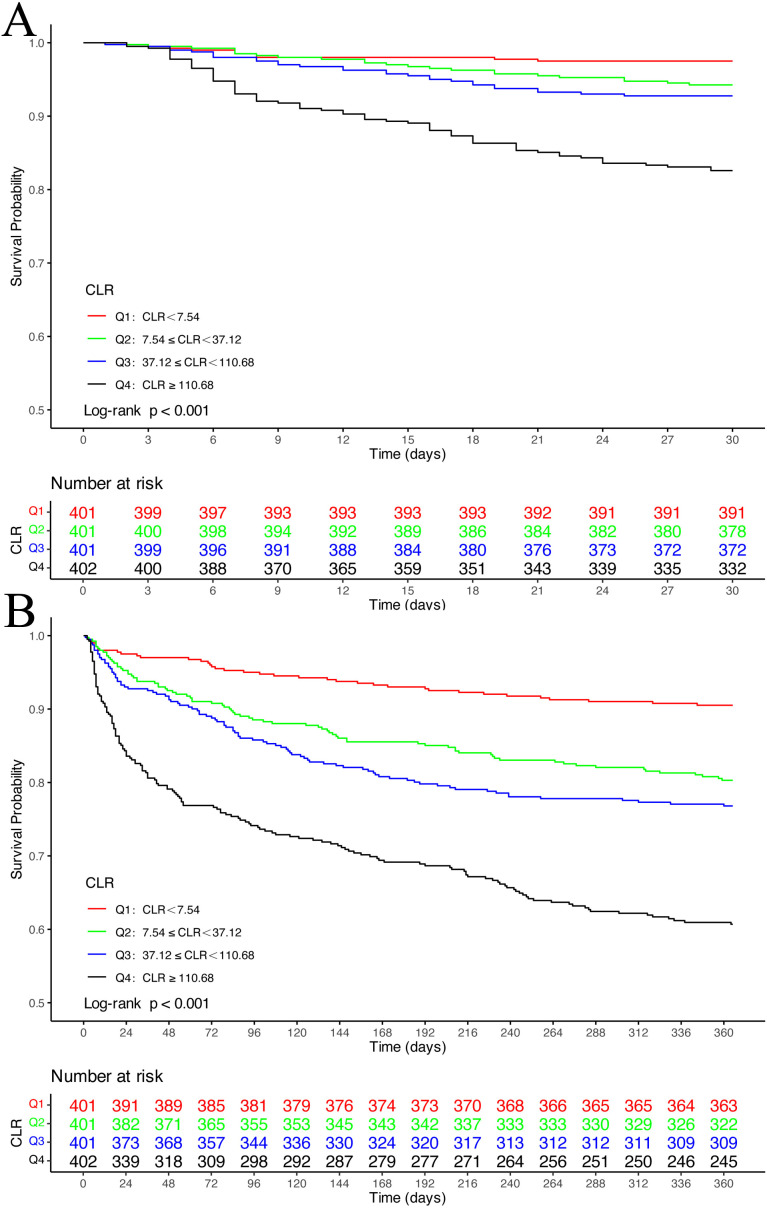
Kaplan-Meier survival analysis curves for all-cause mortality. Kaplan-Meier curves of 30-day **(A)** and 365-day **(B)** all-cause mortality stratified by CLR index.

The analysis demonstrated significant mortality disparities across CLR quartiles. Patients in the highest CLR quartile (Q4) showed substantially elevated mortality rates at both time points compared to lower quartiles. At 30 days, mortality rates progressively increased from Q1 (2.49%) to Q4 (17.41%) (P < 0.001). This trend continued through 365 days, with Q4 mortality reaching 39.30% versus 9.48% in Q1 (P < 0.001). Corresponding survival analyses (**Figure 2**) confirmed significantly worse outcomes for Q4 patients at both 30-day and 365-day evaluations compared to lower quartile groups (log-rank P < 0.05).

### Relationship between CLR and clinical outcomes of heart failure patients with AKI

To evaluate the independent prognostic value of the CLR index for all-cause mortality, we constructed multivariable Cox proportional hazards regression models with sequential adjustment (**Table 2** for 30-day mortality and **Table 3** for 365-day mortality). For 30-day mortality, in the crude model (Model 1), compared with the lowest CLR quartile (Q1, reference), the HRs (95% CIs) for Q2, Q3, and Q4 were 2.32 (1.10–4.87), 2.96 (1.44–6.07), and 7.52 (3.87–14.58), respectively. After adjustment for demographic variables (Model 2: age, sex, weight, language, marital status, and race), the corresponding HRs were 2.27 (1.08–4.78), 2.69 (1.31–5.54), and 6.79 (3.48–13.24). In the fully adjusted model (Model 3), which further accounted for comorbidities, ICU severity scores (APS III, SAPS II, OASIS), and treatment-related variables (corticosteroid use, vasoactive agents, CRRT), the HRs for Q2, Q3, and Q4 were 1.87 (0.88–3.95), 1.86 (0.89–3.88), and 4.32 (2.19–8.53), respectively. Although the HRs for Q2 and Q3 did not reach statistical significance in Model 3, the P for trend across quartiles remained highly significant (< 0.001). When CLR was modeled as a continuous variable after natural logarithm (ln) transformation, each one-unit increase in LnCLR was associated with a 1.60-fold (95% CI: 1.43–1.80), 1.56-fold (1.39–1.75), and 1.39-fold (1.23–1.56) increased risk of 30-day mortality in Models 1, 2, and 3, respectively (all P < 0.001). For 365-day mortality, consistent associations were observed. In Model 1, HRs for Q2, Q3, and Q4 were 2.19 (1.49–3.22), 2.66 (1.82–3.87), and 5.08 (3.56–7.24). In Model 2, the corresponding HRs were 2.26 (1.54–3.34), 2.70 (1.85–3.94), and 4.99 (3.49–7.13). In the fully adjusted Model 3, HRs for Q2, Q3, and Q4 were 1.96 (1.33–2.90), 2.19 (1.49–3.21), and 3.78 (2.63–5.43), respectively (all P < 0.001; P for trend < 0.001). As a continuous variable, each one-unit increase in LnCLR was associated with a 1.40-fold (1.31–1.49), 1.39-fold (1.30–1.48), and 1.30-fold (1.22–1.39) increased risk of 365-day mortality in Models 1, 2, and 3, respectively (all P < 0.001). In the complete-case analysis, the adjusted HRs for 30-day and 365-day mortality were consistent with those from the primary imputation-based analysis. The results are presented in **Supplementary Tables 1** and **2**. In the sensitivity analysis using ROC-derived optimal cut-offs, the association between CLR and mortality remained highly significant across all models. For 30-day mortality, patients with CLR ≥ 86.415 had a significantly higher risk of death compared to those with CLR < 86.415 in the crude model (HR: 3.59; 95% CI: 2.53–5.08; P < 0.001), after demographic adjustment (Model 2: HR: 3.33; 95% CI: 2.34–4.73; P < 0.001), and after full adjustment (Model 3: HR: 2.42; 95% CI: 1.68–3.48; P < 0.001) (**Supplementary Table 3**).For 365-day mortality, using the optimal cut-off of 47.885, patients in the high CLR group consistently demonstrated an increased mortality risk in Model 1 (HR: 2.54; 95% CI: 2.05–3.15; P < 0.001), Model 2 (HR: 2.49; 95% CI: 2.01–3.09; P < 0.001), and Model 3 (HR: 2.06; 95% CI: 1.66–2.57; P < 0.001) (**Supplementary Table 4**). These results confirm the robustness of the CLR–mortality association across different categorization methods. The ROC-derived cut-offs offer potential reference values for risk stratification, although their clinical applicability requires confirmation through external validation studies in independent cohorts.

**Table 2:** Cox proportional hazard models for 30-day all-cause mortality.

Variables	Model 1		Model 2		Model 3	
	HR (95%CI)	*P*	HR (95%CI)	*P*	HR (95%CI)	*P*
LnCLR	1.60 (1.43 ~ 1.80)	**<.001**	1.56 (1.39 ~ 1.75)	**<.001**	1.39 (1.23 ~ 1.56)	**<.001**
CLR 4 group						
1	1.00 (Reference)		1.00 (Reference)		1.00 (Reference)	
2	2.32 (1.10 ~ 4.87)	**0.026**	2.27 (1.08 ~ 4.78)	**0.031**	1.87 (0.88 ~ 3.95)	0.102
3	2.96 (1.44 ~ 6.07)	**0.003**	2.69 (1.31 ~ 5.54)	**0.007**	1.86 (0.89 ~ 3.88)	0.098
4	7.52 (3.87 ~ 14.58)	**<.001**	6.79 (3.48 ~ 13.24)	**<.001**	4.32 (2.19 ~ 8.53)	**<.001**
HR for trend	1.01 (1.01 ~ 1.01)		1.01 (1.01 ~ 1.01)		1.01 (1.01 ~ 1.01)	
*P* for trend		**<.001**		**<.001**		**<.001**

Bold values P < .005 indicates statistical significance.

**Table 3:** Cox proportional hazard models for 365-day all-cause mortality.

Variables	Model 1		Model 2		Model 3	
	HR (95%CI)	*P*	HR (95%CI)	*P*	HR (95%CI)	*P*
LnCLR	1.40 (1.31 ~ 1.49)	**<.001**	1.39 (1.30 ~ 1.48)	**<.001**	1.30 (1.22 ~ 1.39)	**<.001**
CLR 4 group						
1	1.00 (Reference)		1.00 (Reference)		1.00 (Reference)	
2	2.19 (1.49 ~ 3.22)	**<.001**	2.26 (1.54 ~ 3.34)	**<.001**	1.96 (1.33 ~ 2.90)	**<.001**
3	2.66 (1.82 ~ 3.87)	**<.001**	2.70 (1.85 ~ 3.94)	**<.001**	2.19 (1.49 ~ 3.21)	**<.001**
4	5.08 (3.56 ~ 7.24)	**<.001**	4.99 (3.49 ~ 7.13)	**<.001**	3.78 (2.63 ~ 5.43)	**<.001**
HR for trend	1.01 (1.01 ~ 1.01)		1.01 (1.01 ~ 1.01)		1.01 (1.01 ~ 1.01)	
*P* for trend		**<.001**		**<.001**		**<.001**

Bold values P < .005 indicates statistical significance.

**Figure 3:**
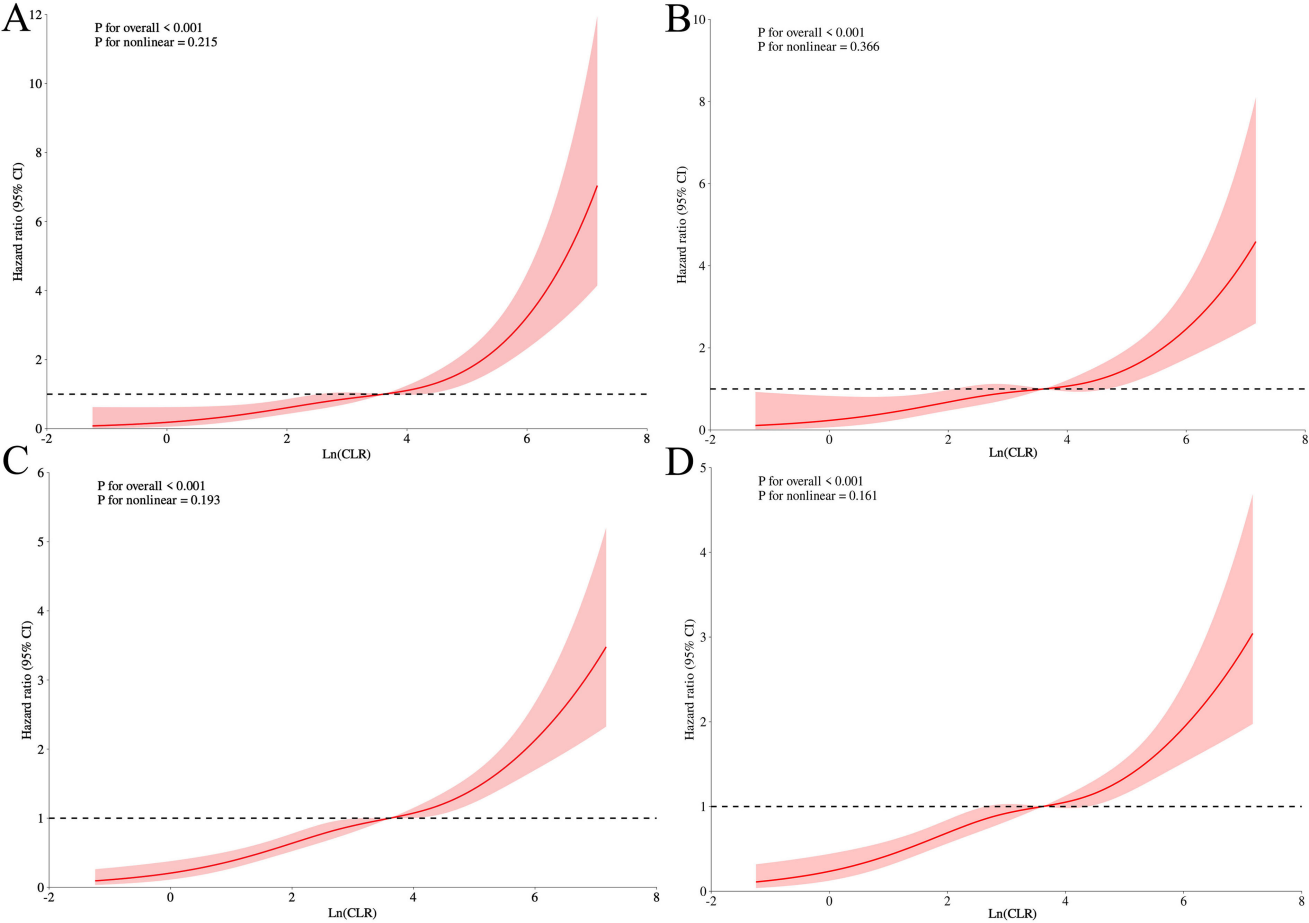
RCS (Restricted Cubic Splines) of Ln (CLR) index with all-cause mortality. RCS of ALI index for 30-day mortality without covariate adjustment **(A)** and with covariate adjustment **(B)**. RCS of ALI index for 365-day mortality without covariate adjustment **(C)** and with covariate adjustment **(D)**.

To flexibly examine the shape of the association, we fitted RCS Cox regression using ln-transformed CLR with four knots placed at the 5th, 35th, 65th, and 95th percentiles. After full adjustment for the same covariates as in Model 3, a linear dose-response relationship was observed between LnCLR and both 30-day and 365-day mortality (P for nonlinearity = 0.352 and 0.197, respectively; P for overall < 0.001 for both), as illustrated in **Figure 3**. These findings demonstrate that elevated CLR levels are independently and robustly associated with an increased risk of both short-term and long-term all-cause mortality in patients with heart failure and acute kidney injury. The association is linear, dose-dependent, and persists after rigorous adjustment for demographics, comorbidities, illness severity, and key ICU treatments.

### analyse Stratified s

**Figure 4:**
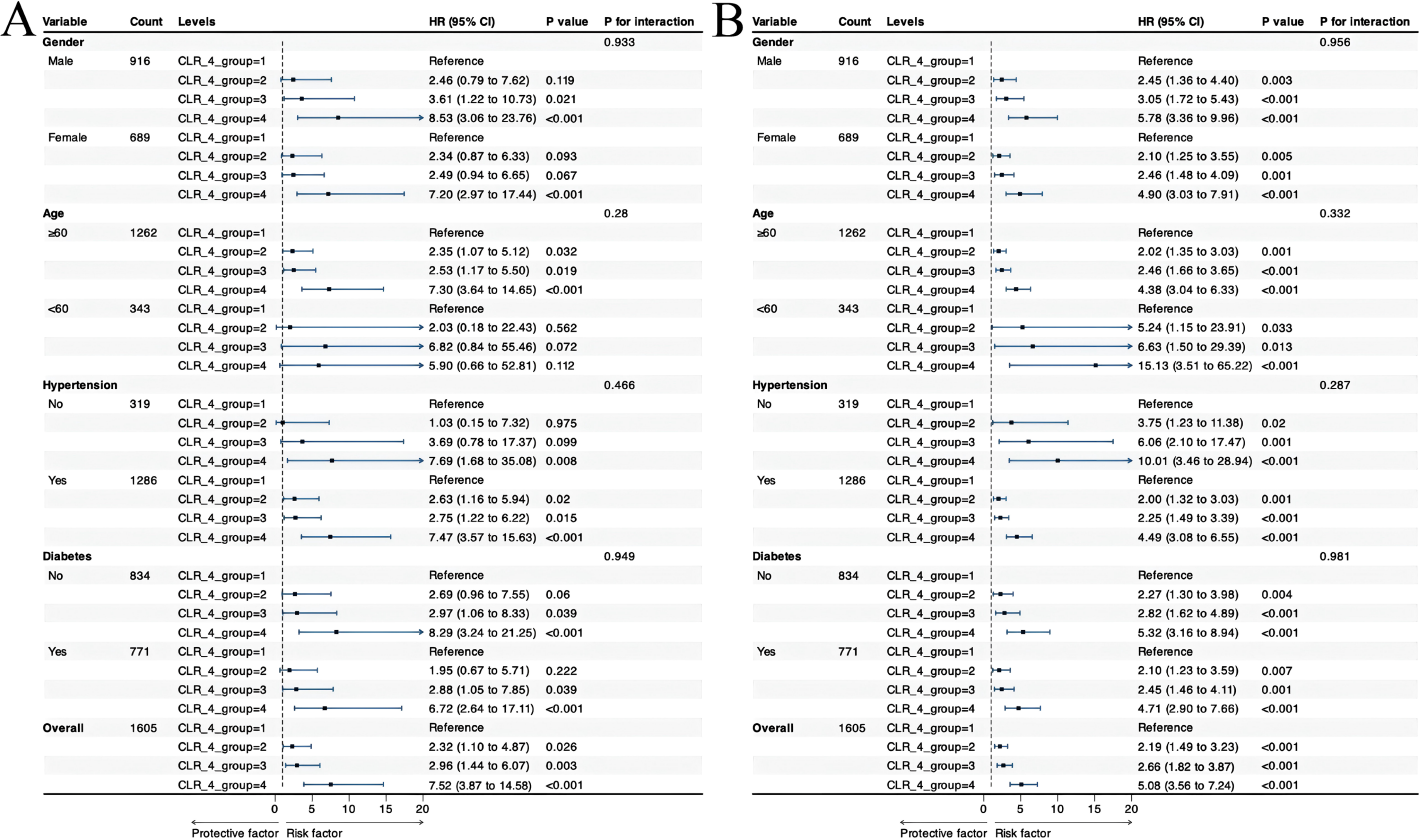
Forest plots of stratified analyses showing the relationship between CLR index with 30-day **(A)** and 365-day **(B)** all-cause mortality.

Subgroup analyses elucidated a consistent risk trend across various demographics and health conditions (gender, age, hypertension, diabetes), with individuals in the highest CLR quartile (CLR_4 group = 4) experiencing significantly elevated hazard ratios (HRs), consistently above 5.9, all achieving statistical significance (P < 0.001). The examination of subgroup variances revealed gender-specific risks; men in the CLR_4 group = 3 faced a notable risk with an HR of 3.61 (P = 0.021), while women showed a trend towards significance at this level (P = 0.067). Age also influenced risk profiles, with patients aged ≥60 years demonstrating significant HRs across all CLR categories, in contrast to those under 60, who exhibited non-significant results, likely due to a smaller sample size. Hypertension emerged as a significant modifier; hypertensive patients displayed notable HRs from CLR_4 group = 2 through 4, whereas no significant relationships were observed in non-hypertensive individuals. In terms of diabetes status, the highest CLR group (CLR_4 group = 4) was significantly linked to increased risk in both diabetic and non-diabetic groups, though non-diabetic patients showed borderline significance for CLR_4 group = 2 (P = 0.06). Additionally, interaction tests revealed no significant differences across these subgroups, with all P-values for interaction above 0.05, indicating that factors such as gender, age, hypertension, and diabetes do not alter the association between high CLR levels and outcomes (**Figure 4**).

### analyse ROC s

**Figure 5:**
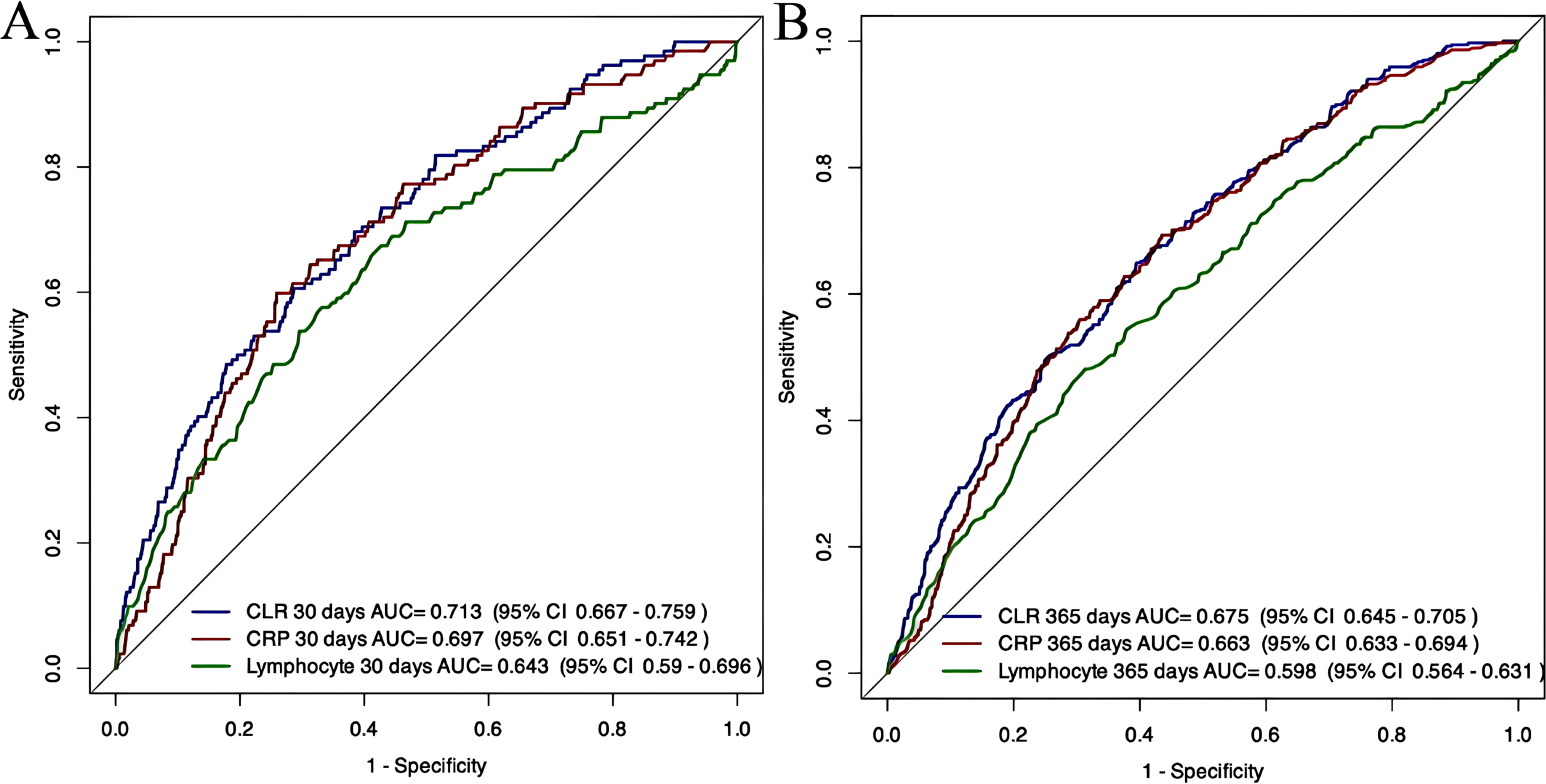
ROC curves for CLR, CRP, and lymphocyte predictive performance at 30-day **(A)** and 365-Day **(B)**.

**Figure 5** presents the receiver operating characteristic (ROC) analysis outcomes. At 30-day evaluation, the CLR demonstrated an area under the curve (AUC) of 0.713 (95% confidence interval [CI]: 0.667-0.759), outperforming both CRP (CRP; AUC = 0.697, 95% CI: 0.651-0.742) and lymphocyte count (AUC = 0.643, 95% CI: 0.590-0.696). Statistical comparison using DeLong’s method revealed comparable predictive performance between CLR and CRP (p=0.247), whereas CLR showed superior discrimination capability versus lymphocyte parameters (p=0.003). No significant difference emerged between CRP and lymphocyte measures (p=0.121). For long-term prognosis assessment (365-day), the CLR maintained its predictive advantage with an AUC of 0.675 (95% CI: 0.645-0.705), compared to CRP’s 0.663 (95% CI: 0.633-0.694) and lymphocyte’s 0.598 (95% CI: 0.564-0.631). DeLong’s test confirmed CLR’s statistically significant superiority over lymphocyte measurement (p<0.001) and CRP’s advantage over lymphocyte count (p=0.003), while CLR and CRP showed comparable performance (p=0.15). These findings establish CLR as a clinically relevant prognostic indicator with enhanced predictive capacity compared to isolated lymphocyte evaluation, particularly evident in both short-term and extended mortality prediction contexts.

## Discussion

The investigation establishes CLR as a reliable standalone indicator of clinical outcomes in patients with acute cardiorenal syndrome. Our data showed a clear concentration–response relationship: as CLR levels progressively rose, mortality risk increased exponentially. Specifically, after full adjustment for demographics, comorbidities, illness severity scores, and key ICU interventions (Model 3), subjects in the highest CLR quartile (≥110.68 units) had a 4.32-fold (95% CI: 2.19–8.53) increased risk of 30-day mortality and a 3.78-fold (95% CI: 2.63–5.43) increased risk of 365-day mortality compared with those in the lowest quartile. This prognostic consistency remained statistically significant after rigorous multivariate adjustments—including Cox proportional hazards modeling and propensity score matching. Sensitivity analyses across demographic subgroups further confirmed CLR’s clinical relevance in multi-organ failure settings. These findings align with recent critical care literature and extend CLR’s applicability to cardiorenal pathophysiology, where synergistic organ dysfunction amplifies both inflammatory cascades and immune dysregulation (Tang, 2020; Zhang et al., 2021).

CLR’s predictive strength may stem from its dual capacity to reflect systemic inflammation (via elevated CRP) and adaptive immune compromise (via lymphopenia). Hepatic CRP, driven by IL-6 signaling, exacerbates vascular endothelial damage through redox imbalance and promotes fibrotic remodeling in myocardial and renal tissues (Burger et al., 2023; Boulet et al., 2024). Lymphocytopenia in cardiorenal syndrome has multiple possible causes. These include corticosteroid-induced apoptosis, inflammatory cytokine-mediated suppression of hematopoiesis, and lymphocyte sequestration in edematous organs (Liu et al., 2020; Liu et al., 2022). Thus, CLR captures a vicious cycle: chronic inflammation leads to immune paralysis, which raises infection risk and impairs tissue repair. Supporting this, sepsis models confirm that CLR correlates with dysregulated neutrophil activity and hyperinflammation—processes also central to cardiorenal injury (Silvestre-Roig et al., 2020; Chen et al., 2021).

ROC curve assessments confirmed CLR’s diagnostic superiority over isolated lymphocyte indices (AUC 0.713 versus 0.643, p<0.01) for mortality prediction, underscoring the clinical advantage of combined biomarker evaluation. While CRP alone demonstrated comparable discriminative power (AUC 0.697), the monotonic CLR-mortality association validated by cubic spline analysis suggests its enhanced capacity to quantify pathophysiological progression. These findings reinforce the emerging paradigm of multidimensional biomarker utilization exemplified by NLR (neutrophil-lymphocyte ratio) and SII (systemic inflammation index) in complex disease states (Yang et al., 2021; Mosca et al., 2023). From a clinical perspective, the consistent prognostic value of CLR supports its potential as a triage tool: patients with CLR ≥110.68 units (Q4) may warrant closer observation and earlier aggressive management of both inflammatory and immunocompromised states, whereas those in lower quartiles could be considered for less intensive monitoring pathways. Notably, although NLR>4.0 predicts adverse outcomes in CHF cohorts (HR = 2.14 for 12-month mortality), its prognostic utility in AKI-dominated populations requires further validation (Wu et al., 2023). Our results position CLR as a particularly sensitive indicator for inflammation-immunity crosstalk in cardiorenal pathophysiology.

Stratified analyses maintained CLR’s prognostic validity across most demographic and comorbidity categories, though diminished predictive performance emerged in younger cohorts (<60 years) and normotensive subgroups. This attenuation may reflect either limited statistical power in smaller subgroups or distinct pathobiological mechanisms - potentially including preserved immune adaptive capacity in younger patients versus accentuated endothelial pathology in hypertension-mediated organ damage (Matsushita et al., 2022, 29). These differential patterns highlight the necessity for age-specific risk stratification protocols and motivate longitudinal studies with repeated CLR measurements to capture dynamic risk evolution.

Several methodological constraints warrant consideration. First, the observational design introduces potential selection bias and residual confounding from undocumented variables, particularly regarding immunosuppressive therapies and anti-inflammatory regimens that may modulate CLR trajectories. Second, the exclusive reliance on a single-center ICU database (MIMIC-IV) significantly limits the generalizability of our findings. This restriction affects external validity not only due to institution-specific clinical protocols and regional epidemiological factors, but also because of potential differences in patient demographics, comorbidities, and disease severity spectra compared to non-ICU settings or other geographic populations—especially non-Western healthcare systems. Variability in CRP measurement practices and calibration across institutions may further influence biomarker-outcome associations. Third, our single-time-point assessment of CLR at ICU admission did not capture potential temporal biomarker fluctuations that might improve prognostic accuracy. Further prospective studies are needed to better establish the predictive utility of CLR over time. Fourth, the interpretation of subgroup analyses, especially in smaller cohorts such as patients aged <60 years, requires caution. The lack of significant associations in these groups for short-term (30-day) mortality—in contrast to the strong associations observed for long-term (365-day) outcomes—suggests that statistical power may have been insufficient to detect short-term effects, or that the pathophysiological impact of CLR evolves over time. Therefore, these non-significant short-term findings should be considered inconclusive rather than evidence of no association. Finally, while CLR’s biological plausibility is supported by existing sepsis research, its precise mechanistic role in cardiorenal syndromes requires elucidation through targeted investigations of neutrophil extracellular trap dynamics and cytokine network interactions.

## Conclusion

In conclusion, CLR serves as an accessible and cost-effective biomarker that independently is associated with mortality among CHF patients with AKI. If validated in future prospective multicenter studies, incorporating CLR into risk stratification models may facilitate early identification of high-risk patients and support more personalized management. To further establish the reliability of these findings and explore the mechanisms through which CLR is linked to adverse outcomes in cardiorenal syndromes, additional large-scale, multicenter studies are required.

## Data Availability

The datasets presented in this study can be found in online repositories (in the MIMIC-III database, Record ID: 52795482). The names of the repository/repositories and accession number(s) can be found in the article/**Supplementary Material**.

## Supplementary material

Supplementary Figure 1 Visualization of missing values.

Supplementary Table 1 Cox regression analysis after complete-case deletion (30-day mortality).

Supplementary Table 2 Cox regression analysis after complete-case deletion (365-day mortality).

Supplementary Table 3 Multivariate Cox regression analysis for 30-day prognosis after dichotomization based on cutoff value.

Supplementary Table 4 Multivariate Cox regression analysis for 365-day prognosis after dichotomization based on cutoff value.
