# Supplementary figures and images for: Predictive value of the C-reactive protein-to-lymphocyte ratio for prognosis in heart failure patients with acute kidney injury
Source: Front Physiol. 2026 May 19;17:1746567. doi: 10.3389/fphys.2026.1746567 (PMC13225966; doi:10.3389/fphys.2026.1746567)

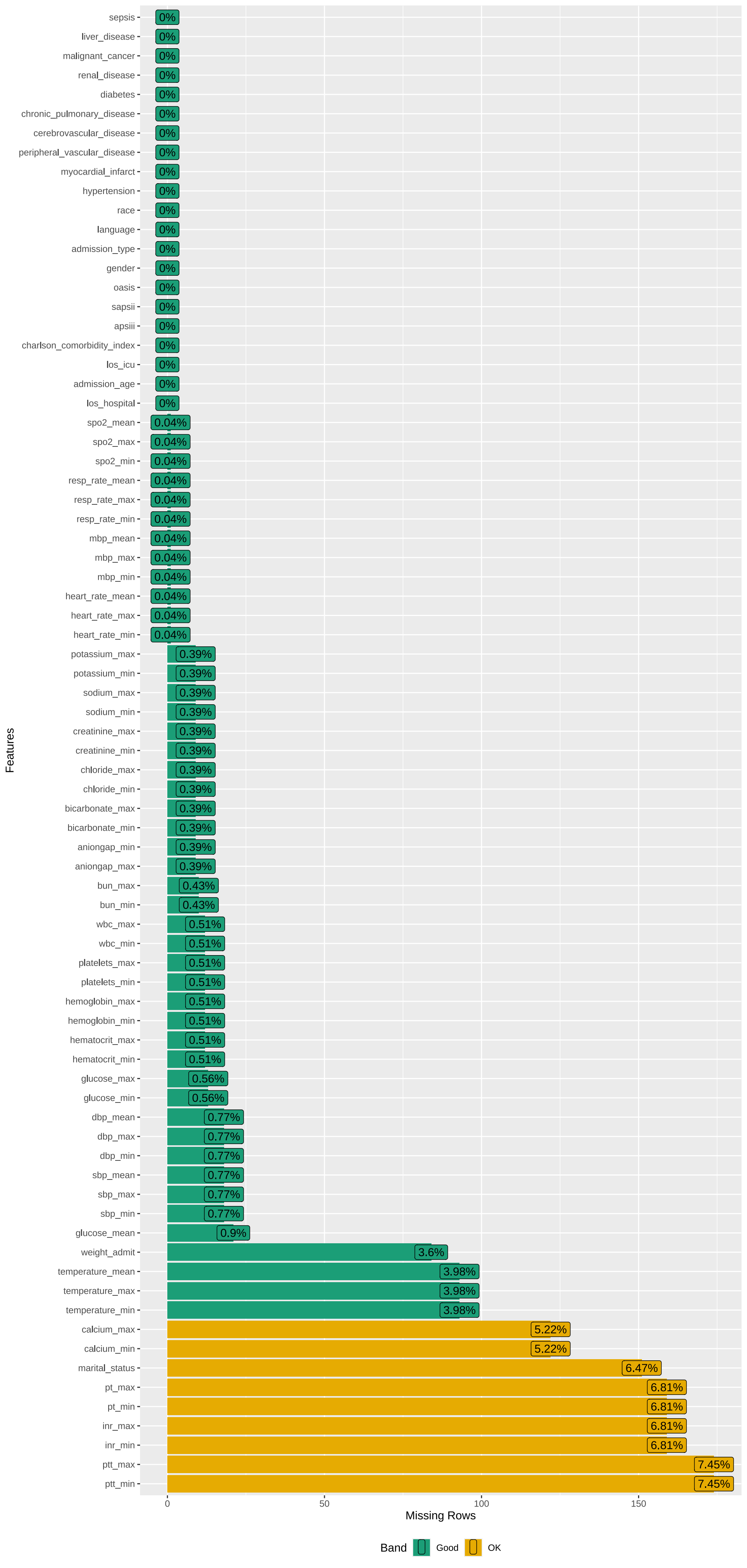

Supplement: Supplementary Figure 1 — Visualization of missing values. [file DataSheet1.pdf]
