# Supplementary material for: Predictive value of the C-reactive protein-to-lymphocyte ratio for prognosis in heart failure patients with acute kidney injury
Source: Front Physiol. 2026 May 19;17:1746567. doi: 10.3389/fphys.2026.1746567 (PMC13225966; doi:10.3389/fphys.2026.1746567)
Supplement: Supplementary Table 1 — Cox regression analysis after complete-case deletion (30-day mortality). [file Table1.docx]

**Supplementary Table 1. Cox regression analysis after complete-case deletion (30-day mortality).**

| Variables | Model1 | |  | Model2 | |  | Model3 | |
| --- | --- | --- | --- | --- | --- | --- | --- | --- |
|  | HR (95%CI) | *P* |  | HR (95%CI) | *P* |  | HR (95%CI) | *P* |
| LnCLR | 1.57 (1.38 ~ 1.79) | **<.001** |  | 1.53 (1.34 ~ 1.75) | **<.001** |  | 1.37 (1.19 ~ 1.58) | **<.001** |
| CLR 4 group |  |  |  |  |  |  |  |  |
| 1 | 1.00 (Reference) |  |  | 1.00 (Reference) |  |  | 1.00 (Reference) |  |
| 2 | 2.37 (1.03 ~ 5.45) | **0.042** |  | 2.30 (1.00 ~ 5.31) | 0.050 |  | 1.88 (0.80 ~ 4.37) | 0.145 |
| 3 | 2.76 (1.22 ~ 6.23) | **0.015** |  | 2.66 (1.17 ~ 6.02) | **0.019** |  | 1.76 (0.77 ~ 4.04) | 0.179 |
| 4 | 7.43 (3.52 ~ 15.65) | **<.001** |  | 6.61 (3.12 ~ 14.00) | **<.001** |  | 4.04 (1.87 ~ 8.73) | **<.001** |
| HR for trend | 1.01 (1.01 ~ 1.01) |  |  | 1.01 (1.01 ~ 1.01) |  |  | 1.01 (1.01 ~ 1.01) |  |
| *P* for trend |  | **<.001** |  |  | **<.001** |  |  | **<.001** |
| HR: Hazard Ratio, CI: Confidence Interval | | | | | | | | |
| Model1: Crude | | | | | | | | |
| Model2: Adjust: gender, language, marital_status, race, admission_age, weight_admit | | | | | | | | |
| Model3: Adjust: gender, language, marital_status, race, hypertension, myocardial_infarct, peripheral_vascular_disease, cerebrovascular_disease, chronic_pulmonary_disease, diabetes, renal_disease, malignant_cancer, liver_disease, corticosteroids, vasoactive_used, CRRT, admission_age, apsiii, sapsii, oasis, weight_admit | | | | | | | | |
|  | | | | | | | | |
