# Supplementary material for: Predictive value of the C-reactive protein-to-lymphocyte ratio for prognosis in heart failure patients with acute kidney injury
Source: Front Physiol. 2026 May 19;17:1746567. doi: 10.3389/fphys.2026.1746567 (PMC13225966; doi:10.3389/fphys.2026.1746567)
Supplement: Supplementary Table 2 — Cox regression analysis after complete-case deletion (365-day mortality). [file Table2.docx]

**Supplementary Table 2. Cox regression analysis after complete-case deletion (365-day mortality).**

| Variables | Model1 | |  | Model2 | |  | Model3 | |
| --- | --- | --- | --- | --- | --- | --- | --- | --- |
|  | HR (95%CI) | *P* |  | HR (95%CI) | *P* |  | HR (95%CI) | *P* |
| LnCLR | 1.37 (1.28 ~ 1.47) | **<.001** |  | 1.37 (1.27 ~ 1.46) | **<.001** |  | 1.29 (1.20 ~ 1.38) | **<.001** |
| CLR 4 group |  |  |  |  |  |  |  |  |
| 1 | 1.00 (Reference) |  |  | 1.00 (Reference) |  |  | 1.00 (Reference) |  |
| 2 | 2.25 (1.49 ~ 3.39) | **<.001** |  | 2.31 (1.53 ~ 3.49) | **<.001** |  | 1.99 (1.32 ~ 3.02) | **0.001** |
| 3 | 2.62 (1.76 ~ 3.92) | **<.001** |  | 2.74 (1.83 ~ 4.11) | **<.001** |  | 2.15 (1.43 ~ 3.23) | **<.001** |
| 4 | 4.87 (3.33 ~ 7.11) | **<.001** |  | 4.74 (3.23 ~ 6.94) | **<.001** |  | 3.59 (2.44 ~ 5.30) | **<.001** |
| HR for trend | 1.01 (1.01 ~ 1.01) |  |  | 1.01 (1.01 ~ 1.01) |  |  | 1.01 (1.01 ~ 1.01) |  |
| *P* for trend |  | **<.001** |  |  | **<.001** |  |  | **<.001** |
| HR: Hazard Ratio, CI: Confidence Interval | | | | | | | | |
| Model1: Crude | | | | | | | | |
| Model2: Adjust: gender, language, marital_status, race, admission_age, weight_admit | | | | | | | | |
| Model3: Adjust: gender, language, marital_status, race, hypertension, myocardial_infarct, peripheral_vascular_disease, cerebrovascular_disease, chronic_pulmonary_disease, diabetes, renal_disease, malignant_cancer, liver_disease, corticosteroids, vasoactive_used, CRRT, admission_age, apsiii, sapsii, oasis, weight_admit | | | | | | | | |
