# Supplementary material for: Predictive value of the C-reactive protein-to-lymphocyte ratio for prognosis in heart failure patients with acute kidney injury
Source: Front Physiol. 2026 May 19;17:1746567. doi: 10.3389/fphys.2026.1746567 (PMC13225966; doi:10.3389/fphys.2026.1746567)
Supplement: Supplementary Table 3 — Multivariate Cox regression analysis for 30-day prognosis after dichotomization based on cutoff value. [file Table3.docx]

**Supplementary Table 3. Multivariate Cox regression analysis for 30-day prognosis after dichotomization based on cutoff value.**

| Variables | Model1 | |  | Model2 | |  | Model3 | |
| --- | --- | --- | --- | --- | --- | --- | --- | --- |
|  | HR (95%CI) | *P* |  | HR (95%CI) | *P* |  | HR (95%CI) | *P* |
| CLR 2 group |  |  |  |  |  |  |  |  |
| ＜86.415 | 1.00 (Reference) |  |  | 1.00 (Reference) |  |  | 1.00 (Reference) |  |
| ≥86.415 | 3.59 (2.53 ~ 5.08) | **<.001** |  | 3.33 (2.34 ~ 4.73) | **<.001** |  | 2.42 (1.68 ~ 3.48) | **<.001** |
| HR: Hazard Ratio, CI: Confidence Interval | | | | | | | | |
| Model1: Crude | | | | | | | | |
| Model2: Adjust: gender, language, marital_status, race, admission_age, weight_admit | | | | | | | | |
| Model3: Adjust: gender, language, marital_status, race, hypertension, myocardial_infarct, peripheral_vascular_disease, cerebrovascular_disease, chronic_pulmonary_disease, diabetes, renal_disease, malignant_cancer, liver_disease, corticosteroids, vasoactive_used, CRRT, admission_age, apsiii, sapsii, oasis, weight_admit | | | | | | | | |
|  | | | | | | | | |
